# Supplementary material for: Rollout of the 2022/2023 Seasonal Influenza Vaccination and Correlates of the Use of Enhanced Vaccines among Italian Adults
Source: Vaccines (Basel). 2023 Nov 23;11(12):1748. doi: 10.3390/vaccines11121748 (PMC10747154; doi:10.3390/vaccines11121748)
Supplement: Supplementary file 1 [file vaccines-11-01748-s001.zip › vaccines-2730744-supplementary.pdf]

Table S1. Multivariable logistic regression models [1] to predict uptake of the 2022/23 seasonal influenza vaccination among adults aged ≥ 60 years.

Variable	Level	aOR (95% CI) [2]	aOR (95% CI) [3]
Sex	Male	–	Ref
	Female	–	1.09 (0.94–1.29)
Age, years	1-year increase	1.02 (1.01–1.03)	1.02 (1.01–1.03)
Chronic conditions [4]	Cardiovascular	2.04 (1.74–2.40)	2.03 (1.73–2.38)
	Diabetes	1.41 (1.13–1.75)	1.56 (1.12–2.21)
	Respiratory	1.40 (0.91–2.18)	1.39 (0.90–2.17)
	Renal	–	1.27 (0.87–1.88)
	Hepatic	2.77 (1.38–6.04)	2.83 (1.41–6.19)
	Rheumatic	0.58 (0.33–1.05)	0.57 (0.32–1.03)
	Cancer and immunosuppressive	1.62 (1.26–2.09)	1.60 (1.24–2.06)
	Anemia	1.44 (1.10–1.88)	1.41 (1.08–1.85)
	Obesity	1.45 (0.94–2.29)	1.45 (0.93–2.29)
	Dementia	2.67 (0.90–9.75)	2.67 (0.91–9.77)

[1] Models are also adjusted for general practitioner; [2] Selection of variables in the multivariable model based on minimization of the Akaike information criterion; [3] Fully adjusted model; [4] Reference category is set to the absence of a given condition; aOR, adjusted odds ratio; CI, confidence interval.
